# Travel distance and sociodemographic correlates of potentially avoidable emergency department visits in California, 2006–2010: an observational study

**DOI:** 10.1186/s12939-015-0158-y

**Published:** 2015-03-21

**Authors:** Brian K Chen, James Hibbert, Xi Cheng, Kevin Bennett

**Affiliations:** Department of Health Services Policy and Management, Arnold School of Public Health, University of South Carolina, 915 Greene Street Suite 354, Columbia, SC 29208 USA; Center for Research in Nutrition and Health Disparities, University of South Carolina, 921 Assembly Street #230, Columbia, SC 29208 USA; Arnold School of Public Health, University of South Carolina, 915 Greene Street, Columbia, SC 29208 USA; Family and Preventive Medicine, University of South Carolina School of Medicine, 3209 Colonial Dr., Columbia, SC 29203 USA

**Keywords:** Avoidable emergency department utilization, NYU ED Algorithm, Barriers to primary care, Medicaid, Vulnerable populations, Health disparities

## Abstract

**Introduction:**

Use of the hospital emergency department (ED) for medical conditions not likely to require immediate treatment is a controversial topic. It has been faulted for ED overcrowding, increased expenditures, and decreased quality of care. On the other hand, such avoidable ED utilization may be a manifestation of barriers to primary care access.

**Methods:**

A random 10% subsample of all ED visits with unmasked variables, or approximately 7.2% of all ED visits in California between 2006 and 2010 are used in the analysis. Using panel data methods, we employ linear probability and fractional probit models with hospital fixed effects to analyze the associations between avoidable ED utilization in California and observable patient characteristics. We also test whether shorter estimated road distances to the hospital ED are correlated with non-urgent ED utilization, as defined by the New York University ED Algorithm. We then investigate whether proximity of a Federally Qualified Health Center (FQHC) is correlated with reductions in non-urgent ED utilization among Medicaid patients.

**Results:**

We find that relative to the reference group of adults aged 35–64, younger patients generally have higher scores for non-urgent conditions and lower scores for urgent conditions. However, elderly patients (≥65) use the ED for conditions more likely to be urgent. Relative to male and white patients, respectively, female patients and all identified racial and ethnic minorities use the ED for conditions more likely to be non-urgent. Patients with non-commercial insurance coverage also use the ED for conditions more likely to be non-urgent. Medicare and Medicaid patients who live closer to the hospital ED have higher probability scores for non-emergent visits. However, among Medicaid enrollees, those who live in zip codes with an FQHC within 0.5 mile of the zip code population centroid visit the ED for medical conditions less likely to be non-emergent.

**Conclusions:**

These patterns of ED utilization point to potential barriers to care among historically vulnerable groups, observable even when using rough estimates of travel distances and avoidable ED utilization.

**Electronic supplementary material:**

The online version of this article (doi:10.1186/s12939-015-0158-y) contains supplementary material, which is available to authorized users.

## Introduction

Use of the hospital emergency department (ED) for avoidable medical conditions is often faulted for waste and inefficiency, but may be a symptom of challenges in access to health care [1]. It is a controversial topic, given the potentially high cost and negative consequences of avoidable ED utilization. It is estimated that avoidable ED visits cost $38 billion annually in the United States [2], a figure that is likely far greater if subsequent intensive or inpatient care could have been averted through access to appropriate and timely primary care [3].

Given its cost and health implications, there has been substantial policy and academic interest in avoidable ED utilization. Research attempting to identify the patterns of avoidable ED visits dates back four decades [4,5], and efforts to curb their occurrence go back to the Johnson administration [1]. Recent studies predict that the Affordable Care Act will further increase ED usage [6], intensifying the pressure to address the looming healthcare crisis of increasing costs and reduced accessibility to care. In particular, the episodic and reactive nature of ED medical care makes it ill-suited as a medical home for chronically ill patients. It is therefore of great policy importance to understand the correlates of avoidable ED utilization, particularly at the population level in the most populous state in the United States.

Despite over four decades of research, debate continues regarding the extent, causes, and consequences of avoidable ED utilization. Moreover, only limited research exists to offer insight as to how *types* of avoidable ED utilization differ by socioeconomic groups, a necessary first step to understand the distribution of medical need in the population. Avoidable utilization encompasses a wide range of reasons, including conditions that likely do not require immediate treatment, conditions that could be treated in alternative settings, and conditions that could have been prevented with timely and appropriate primary care. In addition, while offering valuable insights and depth of analysis, many existing studies may not be externally valid at a population level. Many do not draw from representative samples, often have study samples smaller than 500 individuals, or apply only bivariate or descriptive statistics [7]. As a result, the literature is inconsistent as to association of various demographic factors and avoidable ED utilization [8-14].

To identify the demographic and geographic distribution of non-emergent, primary care treatable, and primary care preventable ED visits, we identify associations between observable patient characteristics and ED visits with higher probability scores of avoidable ED utilization, using 7.2% of all ED visits in California between 2006 and 2010. In our analysis, we define measures of avoidable ED utilization according to the New York University ED Algorithm [15]. We then investigate whether travel distances to the ED are associated with avoidable ED utilization, and whether Medicaid patients’ avoidable ED utilization patterns differ when a Federally Qualified Health Center (FQHC) exists within half a mile of patients’ zip code population centroid.

Our goal is to offer policymakers an overview of the patterns of avoidable ED utilization at the state level as a first step toward crafting appropriate evidence-based policy responses to this longstanding health care challenge in California.

## Methods

### Data

We use the 2006–2010 Emergency Department public dataset from California’s Office of Statewide Health Planning and Development Office. The source data include 100% of outpatient encounters in all licensed emergency departments and ambulatory surgical centers reported through the Medical Information Reporting for California System.

Data elements reported consist of patient demographic and clinical information, such as age, sex, zip code of residence, race/ethnicity, diagnostic information, disposition, quarter/year of the ED visit, and expected source of payment. However, no information on costs or charges is provided.

We exclude observations of patients who were not residents of California on the date of their ED visit, and observations with diagnoses that are considered “unclassifiable” according to the NYU ED Algorithm (“Algorithm”). Together, non-residents and non-classifiable visits comprise 28% of the full data of over 54 million records. The types and distribution of the 50 most common diagnoses considered unclassifiable treated in California EDs are presented in Additional file 1: Table S1, and include conditions such as fever, pain, administrative visits, attention to dressings/sutures, and skin diseases. We also drop all observations without the complete set of control variables used in our empirical specifications (age range (5 categories), gender, race/ethnicity, quarter-year of the ED visit, full zip code and insurance coverage type) These variables were masked by the data provider in a specified order in order to prevent the identification of individual patients.

We approximate the distance traveled to receive ED care by calculating the shortest road distance between the exact address of the ED facility and the 2008 population centroid of the patient’s zip code. We similarly determine the shortest distance to the exact address of the nearest FQHC in operation in the quarter of the patient’s ED visit. We obtained the list of FQHCs and their dates of operation from the Health Resource and Services Administration (HRSA) Data Warehouse [16]. From these two estimated distances, we construct two indicator variables that respectively equal to 1 if the distance from the patient’s zip code population centroid to the ED is within the 25% percentile of travel distances, and if the distance to the closest FQHC is within 0.5 mile.

After applying the exclusion criteria, we obtained approximately 39 million observations. However, the number far exceeds our computational resources for the bootstraps required to calculate standard errors in our non-linear models, and required us to take a random 10% subsample of the available data, resulting in a total of 3,912,676 observations in the analytical sample.

### Outcome

The dataset includes the ICD-9CM code (International Classification of Diseases 9^th^ Edition, Clinical Modification) of the principal diagnosis given for the ED visit. We convert each of these diagnosis codes into four probability scores of ED utilization based on the New York University ED Algorithm.

Reviewing over 6,000 ED medical records in New York area hospitals between 1994 and 1999, Billings and colleagues developed the Algorithm to assign each discharge diagnosis code a number representing the percentage of cases that are (1) non-emergent (“NE”)(The patient's medical history and presenting symptoms suggested that immediate medical care was not required within 12 hours), (2) primary care treatable (“PCT”) (treatment was required within 12 hours, but could have been provided safely in a primary care setting), (3) primary care preventable (“PCP”) (ED care was required, but could potentially have been preventable/avoidable if timely and effective ambulatory care had been provided), and (4) emergent (ED care was required within 12 hours, and could not have been avoided). We consider the first two categories (NE, PCT) to be non-urgent.

Using the Algorithm, we assign four numbers between 0 and 1 to each ED visit based on the ICD9 code of the principal diagnosis for these four separate outcome variables – NE, PCT, PCP, and emergent. At the individual encounter level, we consider the NE, PCT, PCP and emergent variables to represent the probability scores for each of these four types of ED utilization. For example, the ICD9 code 784.0 (Headache) is assigned a probability of 0.77 for NE, a probability of 0.09 for PCT, and a probability of 0 for PCP.

### Analysis

We conduct the analyses on a longitudinal dataset using standard econometric panel regression techniques with hospital and year fixed effects to control for unobservable, time-invariant confounding at the hospital and year levels. Because the dependent variables are all fractional values bounded below by 0 and above by 1, linear probability models may generate suspect standard errors and inferences. As a result, we also apply a fractional probit model adapted to panel data to verify the outcomes of our linear specifications [17].

The linear model has the following specification:

1 $$ \mathrm{E}\mathrm{D}\ \mathrm{scor}{\mathrm{e}}_{\mathrm{ijt}} = {\upbeta}_0+\upbeta\ {\mathrm{X}}_{\mathrm{ijt}} + {\upalpha}_{\mathrm{j}} + {\uptheta}_{\mathrm{t}} + {\upvarepsilon}_{\mathrm{ijt}} $$

In the specification above, the covariate vector **X** includes the following categorical control variables: patient age group (age ≤ 1, > 1 and < 18, ≥ 18 and < 35, and ≥ 65), female gender, race indicator variables (Black, Asian, Hispanic, and Other), insurance coverage indicator variables (Medicare, Medicaid, Self-Pay, Worker’s Compensation, and Other Programs), and a categorical variable equal to 1 if the distance to the ED is within the lowest quartile of distances traveled for ED care (“Distance”). We also include all interaction terms between the Distance variable and payer categorical variables (except commercial insurance, which is estimated by the Distance variable alone because it is the omitted category in the interactions). Because all of our covariates are categorical variables, in each group, an omitted category was necessary to avoid perfect collinearity in the variables. These include Age 35 to 64, non-Hispanic White race, male gender, and commercial insurance. Subscript *i* refers to each unique encounter, *j*, to each unique hospital ID, and *t*, to year.

All of our regressions exploit the longitudinal nature of the dataset, and include hospital (α_j_) and year fixed effects (θ_t_). This specification provides the coefficient estimates based on within-hospital and within-year variations in measures of ED utilization, and is a common technique used in the economic literature to remove time-invariant unobserved confounding that is fixed for each individual hospital and year.

All of our dependent variables are fractional values between 0 and 1. Therefore, we also use panel data methods for fractional response variables, which are based on a pooled Bernoulli quasi-Maximum Likelihood Estimator that maximizes the pooled probit log-likelihood [17]. Following Papke and Wooldridge, we add the averages of the explanatory variables by hospital ID to replicate the hospital fixed effects in our linear model to control for the correlation between the fixed effects and the explanatory variables.

The resulting coefficients, however, are not directly comparable to the linear model in (1), and are adjusted using a scaled factor as in Papke and Wooldridge to obtain the Average Population Effects (APEs). These partial effects estimate the probability of a dichotomous explanatory variable switching from 0 to 1 across the sample population. All standard errors are robust to serial dependence and clustered at the hospital level. For the fractional probit model, robust standard errors clustered by hospital ID are bootstrapped using the delta method.

All analyses were performed using Stata version 13.1 (College Station, TX).

## Results and discussion

### Patient sample

From 54,494,382 ED visits, we obtained a sample that includes 39,096,084 ED encounters after excluding observations as described in the data section. However, the bootstrap for the standard errors of the fractional probit model demanded computing power that exceeded our computing resources. We therefore created a random 10% subsample from the full data using the CPU clock state on October 22, 2014 at 9:56 p.m. as the random seed, resulting in a total 3,912,676 observations. A comparison of the full and 10% random subsample shows that the distributions of key variables are virtually alike (Additional file 2: Table S2).

### Descriptive statistics

Between 2006 and 2010, ED patients aged 1 and under, 1–17, 18–34, 35–64 and 65 and over represent respectively 3.8%, 24.6%, 26.3%, 33.7% and 11.5% of the ED patient population in California. Women represent 54.8% of the study sample. White, Black, Hispanic, Asian, and individuals of all other races/ethnicities represent, respectively, 41.9%, 10.3%, 17.2%, 3.2%, and 20.2% of ED users. For the expected source of payment, self-pay patients represent 16.6%, Medicaid patients, 27.9%, Medicare patients, 9.6%, workers’ compensation, 1.4%, commercial patients, 28.3%, and all other insurance types, 5.7% of the study sample (Additional file 2: Table S2).

Average NE, PCT, PCP and emergent probability scores were approximately 23.3%, 26%, 7.5% and 13.1% respectively (Additional file 2: Table S2). Based on the NYU Algorithm, approximately 1 in 4 visits was for care that was either not emergent or could have been effectively treated in a primary care setting during 2006–2010.

Additional file 3: Table S3 shows the top 25 diagnoses considered NE, PCT, and PCP with over 75% probability. Pain (headache, migraine, joint pain), skin conditions (eczema, urticaria) and sinusitis populate the top NE reasons. Acute respiratory infections, bronchitis and painful respiration together account for more PCT ED visits than the next 22 most prevalent conditions combined. Asthma and diabetes-related complications, along with congestive heart failure, top the list of PCP ED visits.

In Additional file 1: Table S1, we list the 50 most common diagnoses determined to be unclassifiable according to the NYU Algorithm. From the full sample, 5,028,168 observations were excluded because they were unclassifiable, and 502,820 observations were excluded from the 10% analytic sample. Fever, digestive disorders, disorders of fluid, attention to dressings/sutures, epilepsy/seizures, encounters for administrative purposes, urinary disorders, unspecified pain, other complications of pregnancy and “other suspected conditions” round up the top ten unclassifiable ED visits. Cumulatively, the top 10 unclassifiable conditions represent 29.2%, and the top 50, 54.1% of the unclassifiable conditions in our full sample.

### Regression results

#### Overall patterns

Our regression results show that relative to adults aged 35 to 64, younger individuals visit the ED for conditions that are more likely to be NE and PCT, but less likely to be PCP or emergent. On the other hand, the pattern is the opposite for elderly adults aged 65 and older, who visit the ED for conditions less likely to be NE or PCT, and more likely to be PCP or emergent. Female patients and non-White patients have medical conditions with higher probabilities of being non-urgent. Self-pay, Medicare, and Medicaid patients also have more non-urgent medical conditions in the ED. Finally, Medicare and Medicaid patients who live closest to the ED visit the hospital ED for conditions with higher NE probability scores. On the other hand, Medicaid patients living in zip codes with a Federally Qualified Health Center within 0.5 mile of the population centroid have reduced NE scores when visiting the ED. We present these results in greater detail in the following paragraphs.

#### Visits for NE medical conditions

In Additional file 4: Table S4, Specifications (1), we present the probability of an NE visit relative to the omitted category. Although we present coefficients for the linear model, fractional probit model, and the scale-factor adjusted Average Population Effects (APE) in the Tables, we will refer to the APE estimates in the discussion of results. We note, however, that the estimates and confidence intervals for the linear model and the APEs are very similar, as noted in Papke and Wooldridge [17].

Blacks, Hispanics, Asians, and all other races/ethnicities have NE probabilities that are respectively 3.00 (S.E. 0.120), 1.90 (S.E. 0.117), 1.57 (S.E. 0.165), and 2.03 (S.E. 0.104) percentage points higher than the reference group of White ED patients. Self-pay, Medicare, and Medicaid patients have approximately 0.65 (S.E. 0.251), 1.31 (S.E. 0.168), and 3.69 (S.E. 0.160) higher NE scores than patients with commercial insurance. For Medicare and Medicaid enrollees, shorter travel distances to the ED are associated with higher NE scores, respectively 0.44 (S.E. 0.240; note that p value <0.1 only) and 0.45 (S.E. 0.222) higher than the respective patients who live further away. However, among Medicaid patients, living in a zip code with an FQHC within 0.5 mile of the population centroid is associated with a 0.38 (S.E. 0.145) point reduction in NE scores (Additional file 5: Table S5, Specification (9)).

#### Visits for PCT medical conditions

A similar picture emerges from the analysis with PCT as the dependent variable (Additional file 4: Table S4, Specifications (2)). All racial/ethnic minority groups visit the ED for conditions that have a 2-3% higher chance of being PCT. Medicaid patients have a 2.9-point (S.E. 0.145) higher PCT probability than patients with private commercial insurance coverage. Unlike the NE analysis, no statistically significant relationship is detected for the travel distance variable for Medicaid patients, but a 0.35 point higher PCT score is detectable (p <0.1 only) for Medicare patients living closest to the ED. For Medicaid patients, no statistically significant relationship is found between PCT scores and short distance (<0.5 mile) to the nearest FQHC from the patients’ zip code population centroid (Additional file 5: Table S5, Specification (10)).

#### Visits for PCP medical conditions

For PCP conditions, again all racial and ethnic minorities have higher PCP scores, ranging from 0.18 (S.E. 0.064) to 1.98 (S.E. 0.072) points higher than White patients. As for the non-urgent measures, Black patients and Medicaid enrollees have among the highest PCP scores, respectively at 1.98 (S.E. 0.072) and 1.38 (S.E. 0.070) points greater than their reference groups.

## Discussion

### Demographics/SES

Our findings that females [18], Blacks [19-21], Hispanics [20,22], other minority groups, Medicaid recipients [23-27] and self-pay patients [28] almost consistently seek care in the hospital ED for more avoidable reasons echo some of the existing literature using diverse sources of data and different methods of ED classification. Unsurprisingly, elderly individuals are more likely to visit the ED for PCP medical condition, as they are more likely to have multiple ambulatory care sensitive conditions [29]. Medicaid patients face barriers to primary care, and likely visit the ED for lower acuity NE and PCT conditions where there is a lack of alternative sources of primary care [30]. Black patients, even controlling for insurance status and age, are far more likely to go to the ED for a PCP medical condition, perhaps due both to barriers to access and to distrust of the healthcare system [31].

### Travel distances

We also find that for Medicare and Medicaid patients with shorter travel distances to the ED, the probability scores for NE are higher by 0.44 to 0.45 percentage point. For Medicaid patients, however, the presence of an FQHC within 0.5 mile of the population centroid is associated with a reduced NE probability of 0.38 point. Although these point estimates appear extremely small, it is likely that socioeconomic, demographic, and geographic characteristics are highly correlated [32], so that it is impossible to isolate the pure effect of travel distances on avoidable ED utilization. Nevertheless, using rough estimates of travel distances and of medical urgency, our specifications show a statistically significant relationship between travel distances and avoidable ED visits for publicly insured patients. When these findings are viewed in isolation, our results are consistent with several interpretations, with different potential policy responses. On the one hand, the higher NE probability scores associated with shorter distances to the ED for Medicare/Medicaid patients point to “convenience” as a primary motivating factor in non-urgent ED utilization. When viewed in light of the literature on health disparities, as well as the finding that proximity to an FQHC is associated with a reduction in NE probability scores for Medicaid patients, an alternative narrative may be that of barriers to access combined with misperceived medical urgency.

Indeed, multiple studies point to a lack of access to primary care as a principal reason for avoidable ED visits [33,34]. Furthermore, numerous studies link health illiteracy and mistaken perceptions of clinical urgency as drivers of ED utilization [35,36]. Convenience is also an oft-cited reason for avoidable ED utilization [37,38]. These three reasons all share the common theme of having a correctly or incorrectly perceived medical need that is difficult to address in alternative treatment settings immediately.

What, then, are potential policy responses? If the interpretation is that barriers to access lie at the root of avoidable ED use, increasing access is a logical response. In fact, research indicates that the most promising intervention to reduce avoidable ED utilization consists of increasing access to primary care [39-43]. This strategy is also one that simultaneously addresses the underlying demand for more convenience (demonstrated by the impact of travel distances), and the need for better coordination of care to reduce episodes resulting from PCP medical conditions (as reflected by patients’ use of the ED for PCT and PCP conditions). Other strategies, including care coordination [44], prior utilization authorization for ED visits [45], triage and transfer at point of care [46,47], follow-up visits at FQHCs [48], as well as patient education [49-51], have shown mixed results. Some interventions that focus primarily on the “convenience” interpretation, such as prior authorization for ED utilization, may in fact exacerbate existing barriers to access or become ineffective because of ethical and legal concerns against denying care [52].

Of course, “improving access to the primary care system” is easier said than done. A recent study noted that certain patients are well aware of alternative community resources such as FQHCs but continue to favor the ED because of poor customer service at the health clinics, wait times nearly as long as the ED, affordability, excessive paperwork, and lack of public transportation compared to large tertiary care centers [53]. Some avoidable ED users also cited FQHC staff’s incompatible cultural values as reasons for avoiding health centers for their routine medical care [53]. Ironically, certain hospitals have begun advertising short ED wait times as a hallmark of quality and service, a practice that may inadvertently further attract avoidable ED utilization [54]. Some hospitals have established fast-track ED triage systems with the result that lower acuity patients receive care sooner than moderate acuity patients [55], a system that in theory may further encourage non-urgent utilization.

Improving access to the primary care system therefore appears to require more than mere physical availability of primary care physicians or community health centers. These providers will likely also have to increase their attractiveness to patients on many dimensions, including more timely access and shorter wait times, more easily accessible locations through public transportation, lower financial burden, better service quality, and increased cultural sensitivity. Moreover, primary care providers must also face “competition” from hospital EDs, which are often conveniently located, open 24 hours, required to treat all patients regardless of ability to pay, and need no prior appointment for access. These are certainly tall orders to fill in an increasingly taxed health care system, particularly for the poor and the underserved. However, these are also likely to be the necessary conditions to establish true medical homes for chronically ill patients requiring the level of on-going care not available in the ED.

Indeed, that Black and Medicaid patients visit the ED for higher PCP scores even after controlling for other observable characteristics should be reason for concern. Particular attention should be paid to policies that could increase treatment access and adherence among Black and Medicaid patients, who are especially vulnerable to requiring ED care for conditions that could have been prevented. With implementation of the Affordable Care Act and insurance coverage set to expand through Medicaid eligibility in California, more than simply the costs of avoidable ED expenditures are at stake. ED expenditures are estimated to represent between 1.9% and 5.8% of total health care expenditures nationwide [56], but receiving only episodic care in the ED for chronic conditions may have significant downstream adverse health consequences. Inclusion of costs related to intensive care and hospitalizations because of the lack of appropriate primary care would certainly greatly exceed what appears to be at stake when looking at the costs generated in the ED alone.

### Limitations

The NYU ED Algorithm was developed using data collected from 6,000 ED medical records at six Bronx, New York hospitals between 1994 and 1999. It has not been proven that the Algorithm can be generalized to California from 2006 to 2010.

Nevertheless, a 2010 study supported the validity of the NYU ED Algorithm in differentiating ED utilization based on the subsequent need for hospitalization and/or mortality risk [57]. We also verify the correlation between the Algorithm scores and inpatient or other care after an ED visit in our sample of patients. We find that higher scores for both NE and PCT conditions are associated with a negative probability of further care (any care but “discharge to home”). There is no correlation between PCP scores and further care, but a higher emergent score is associated with a positive probability of further care (See Additional file 6: Table S6).

We also emphasize that the NYU ED Algorithm is meant to provide measures of avoidable ED utilization rather than to serve as a tool for ED triage. Moreover, our estimates are based on the principal diagnosis and do not capture patients’ perceived urgency prior to their diagnosis. Therefore, we consider our study as one that attempts to describe rough probabilities of the urgency of the patients’ diagnosed (rather than self-perceived) medical conditions.

Another limitation is that we do not have patients’ exact home address or point of departure before traveling to receive ED treatment. To address this concern, we increase the likelihood of approximating the patient’s travel distance by using his or her zip code population (rather than geographic) centroid. We also represent differences in travel distances to the nearest FQHC and to the ED as categorical, rather than continuous, variables. We do so because patient perceptions of travel distance are more likely to be influenced by a range of additional miles traveled, rather than by each additional mile traveled.

Finally, the results of this study are relevant primarily to California. Other states, with a smaller or less diverse population and geographic spread, may face different challenges and require alternative policies regarding avoidable ED utilization.

## Conclusion

In this article, using 7.2% of identifiable in-state ED visits in California between 2006 and 2010, we find evidence consistent with health disparities detectable at the state level. Minority groups are more likely to use the hospital ED for avoidable causes. Black and Medicaid patients are particularly likely to go to the ED for medical conditions that could have been averted through timely and appropriate primary care. Shorter travel distances to the ED are associated with higher scores of non-emergent medical conditions for publicly insured patients. Among Medicaid patients, however, an FQHC close to the patient’s zip code population centroid is associated with reduced probabilities of non-emergent ED utilization. Moving forward, improving access to the primary care system for vulnerable populations may serve both to reduce avoidable ED utilization and to provide medical homes for patients requiring coordinated care for their chronic illnesses. The major challenge lies in delivering this expansion in access in a patient-centered and cost-effective manner.

## Additional files

Additional file 1: Table S1. Most Common Unclassifiable ED Visits. Additional file 2: Table S2. Summary Statistics. Additional file 3: Table S3. Top Diagnoses in California EDs with at least 75% Probability of Avoidability. Additional file 4: Table S4. Correlates of Emergency Department Use (All Insurance Types). Additional file 5: Table S5. Correlates of FQHC Distance and ED Utilization among Medicaid Enrollees. Additional file 6: Table S6. Correlation between Further Care and NYU ED Scores.

## Abbreviations

ED: Emergency Department
FQHC: Federally Qualified Health Center
NE: Non-Emergent
NEPCT: Non-Emergent or Primary Care Treatable
NYU ED Algorithm: New York University Emergency Department Algorithm
PCP: Primary Care Preventable
PCT: Primary Care Treatable
VA: Veterans Affairs
